# Bone tunnel placement influences shear stresses at the coracoid process after coracoclavicular ligament reconstruction: a finite element study and radiological analysis

**DOI:** 10.1007/s00402-022-04382-9

**Published:** 2022-03-28

**Authors:** Benjamin Bockmann, L. Dankl, G. Kucinskaite, A. Kumar, J. J. Timothy, G. Meschke, A. J. Venjakob, T. L. Schulte

**Affiliations:** 1 Department of Orthopaedics and Trauma Surgery, St. Josef Hospital, Ruhr University, Bochum, Germany; 2 Department of Orthopaedics and Traumatology, Medical University of Innsbruck, Innsbruck, Austria; 3 Department of Diagnostic and Interventional Radiology and Nuclear Medicine, St. Josef Hospital, Ruhr University, Bochum, Germany; 4 Swiss Federal Institute of Technology, Zürich, Switzerland; 5 Chair of Materials Science and Testing, Technical University of Munich, Munich, Germany; 6 Institute for Structural Mechanics, Ruhr University, Bochum, Germany; 7 Department of Sports Orthopaedics, St. Vinzenz Hospital, Düsseldorf, Germany

**Keywords:** AC joint repair, Shoulder arthroscopy, Shear stress, Coracoid fracture, Finite element analysis

## Abstract

**Introduction:**

Coracoid fractures after arthroscopic treatment of acromioclavicular (AC) joint separations lead to poor clinical outcomes. In this study, different configurations of bone tunnels in the lateral clavicle and coracoid were examined concerning the amount of stress induced in the coracoid.

**Methods:**

An authentic 3D finite element model of an ac joint was established. Three 2.4 mm bone tunnels were inserted in the lateral clavicle, which were situated above, medially and laterally of the coracoid. Then, two 2.4 mm bone tunnels were inserted in the latter, each simulating a proximal and a distal suture button position. Von Mises stress analyses were performed to evaluate the amount of stress caused in the coracoid process by the different configurations. Then, a clinical series of radiographs was examined, the placement of the clavicle drill hole was analyzed and the number of dangerous configurations was recorded.

**Results:**

The safest configuration was a proximal tunnel in the coracoid combined with a lateral bone tunnel in the clavicle, leading to an oblique traction at the coracoid. A distal bone tunnel in the coracoid and perpendicular traction as well as a proximal tunnel in the coracoid with medial traction caused the highest stresses. Anatomical placement of the clavicle drill hole does lead to configurations with smaller stresses.

**Conclusion:**

The bone tunnel placement with the smallest amount of shear stresses was found when the traction of the suture button was directed slightly lateral, towards the AC joint. Anatomical placement of the clavicle drill hole alone was not sufficient in preventing dangerous configurations.

**Level of evidence:**

Controlled laboratory study.

## Introduction

Injuries of the acromioclavicular (AC) joint are common shoulder lesions in athletes [1]. The incidence is considered to be 3–4 cases among 100,000 persons per year, and their ideal treatment is still discussed controversially [2]. While low-grade lesions without alteration of the coracoclavicular (CC) distance show good results after conservative treatment, high-grade lesions demand surgical stabilization in order to maintain a satisfying level of function [3–5]. Despite the possibility of open AC joint repair using hook plate fixation, arthroscopically assisted procedures have gained popularity recently [6]. A common feature of this technique is the establishment of bone tunnels in the lateral clavicle and the coracoid process, which are then pervaded with sutures and buttons in order to restore the CC distance. However, stress fractures of both the lateral clavicle and the coracoid process have been described [7, 8]. Panarello et al. examined a cohort of 896 patients who were treated for AC joint separation and found 12 post-operative coracoid fractures (1.3%) [9]. While this is a rather low percentage, these fractures can be considered a major complication [10], and should be prevented whenever possible.

Concerning the coracoid process, cadaver studies using bones of donors with higher age identified larger drill holes and eccentric tunnel placement as main risk factors for fractures [11]. These studies provide valuable insights into how the location of the bone tunnel influences the stress induced in the coracoid process. However, cadaver experiments show some disadvantages: for instance, the anatomy of the coracoid can differ significantly between specimen. Upon that, many cadavers show reduced bone quality due to the high age of the deceased donor. A method that can overcome these limitations using approximations is the Finite Element (FE) method [12]. FE analysis is a technique with origins in structural engineering for simulating the response of structures under a variety of loading conditions by replicating the experimental testing procedure in a virtual environment using computers [13, 14].

Using this technology, the current study aims at identifying the ideal placement of bone tunnels in the coracoid process and lateral clavicle to reduce shear stresses in the coracoid. Our hypothesis was that not only the placement of bone tunnels in the coracoid, but also the direction of traction of the suture has a relevant impact on the amount of stresses caused.

## Materials and methods

### Building a 3D mesh model of a glenoid and coracoid

**Fig. 1:**
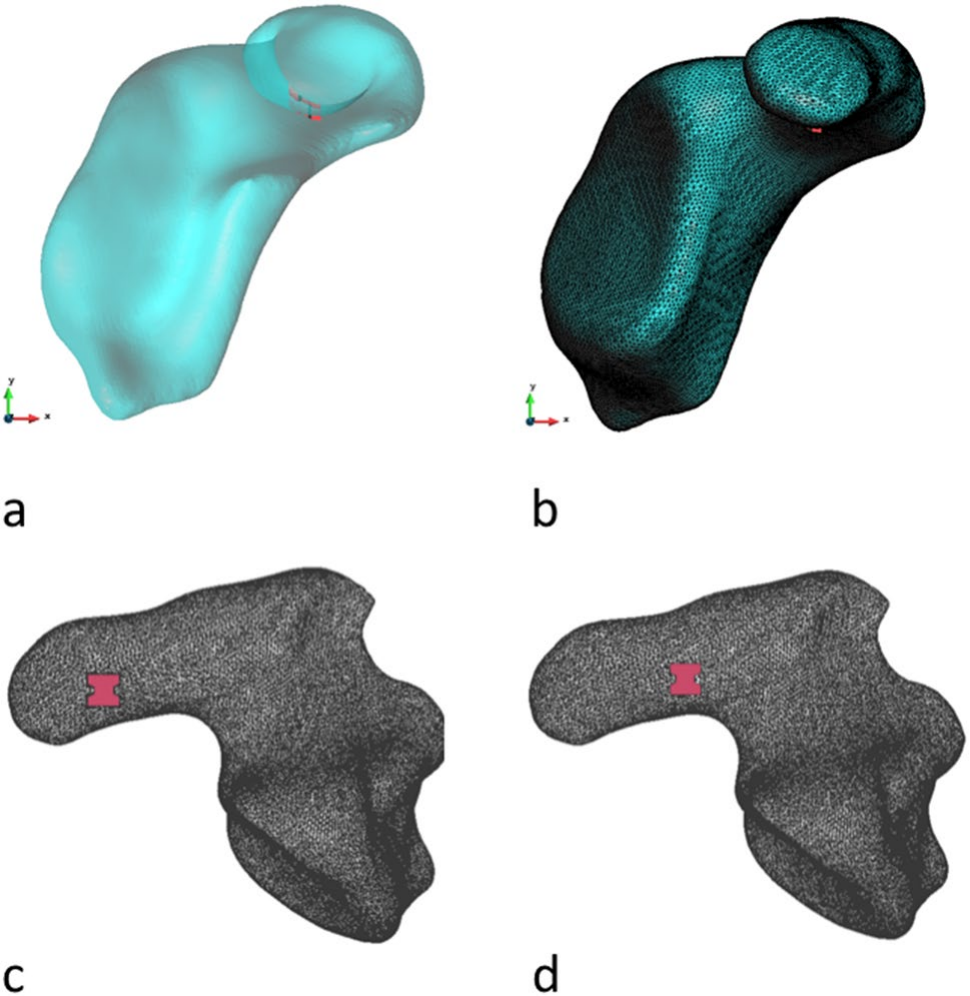
Upper row: The CT model of the glenoid and coracoid was converted to a 3D mesh model (**a**), which was then re-arranged with finite elements (**b**) (parasagittal view). Lower row: Localization of the suture button at the bottom side of the coracoid (axial view). The button was placed at the border between the distal and central third (**c**) and the central and proximal third (**d**) (axial view)

To conduct the analysis, we created a three-dimensional mesh graft model using a CT scan (LightSpeed 63 VCT, GE Healthcare, Chicago, USA; image size 512 × 512, slice thickness 625,000 μm) of a right cadaveric scapula of a 66-year-old male donor. In a next step, the scan was imported into 3D Slicer (Version 4.10.2, http://www.slicer.org), a freeware program designed to create three-dimensional models. A 3D mesh model was created using the manual segmentation function, as described before [15]. The scapula body was sliced off the rest of the bone on the level of the incisura scapulae (Fig. 1a). Edges and irregularities of the model were removed using the Gaussian smoothing algorithm. This 3D model (.stl) of the coracoid process was imported into Gid (http://www.gidhome.com), which is free pre- and post-processing software.

Then, two different bone tunnel locations were virtually placed in the coracoid process (Fig. 1c, d). The diameter of the tunnels was 2.4 mm each, as recommended by the manufacturer of a commonly used system (Dog Bone™ Button Technique, Arthrex^®^, Naples, United States). The first drill hole was located at the border between the distal and the central third (Fig. 1c), the second drill hole at the border between the central and the proximal third of the coracoid body (Fig. 1d).

### Completion of the model by adding a lateral clavicle

**Fig. 2:**
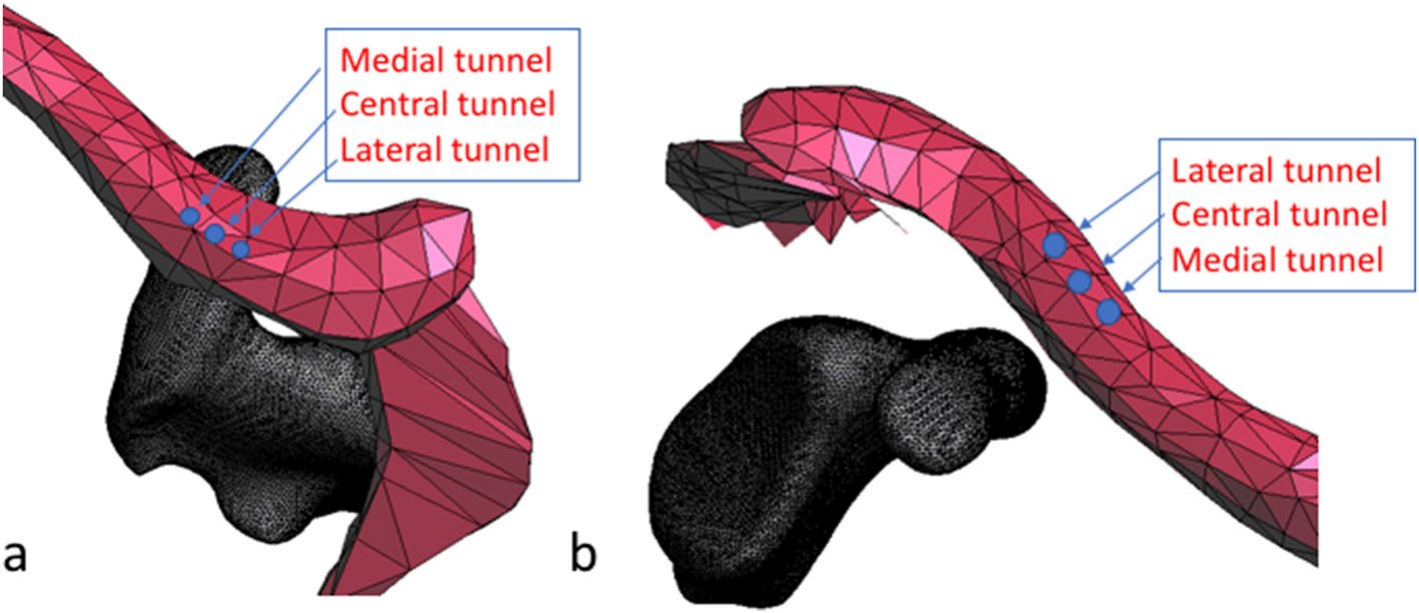
Localization of the bone tunnels in the lateral clavicle, axial (**a**) and oblique (**b**) view

In a next step, a 3D model of a clavicle including the AC joint and acromion from a similar patient was retrieved from a free online database [16]. The two models were fitted to each other (Fig. 2) anatomically by an experienced shoulder surgeon on fellowship level.

Finally, three bone tunnels with a diameter of 2.4 mm were inserted in the lateral clavicle similar to those in the coracoid (Fig. 2a, b). The first tunnel was positioned directly above the coracoid, as confirmed by the three-dimensional axial view. The second and third tunnels were placed 6 mm medially and laterally of the first tunnel, leading to a total of six combinations for the virtual CC repair (two tunnels in the coracoid and three tunnels in the clavicle).

To start biomechanical testing, a virtual suture button was designed according to the specifications of the manufacturer (Dog Bone™ Button, titanium, serial number AR-2270; Arthrex^®^, Naples, United States).

### Finite element analysis and stress evaluation

The complete three-dimensional model of the coracoid process with the virtual suture button was discretized using 420,793 tetrahedral finite elements (Fig. 1b). The clavicle and the bone tunnel were not explicitly part of the finite element analysis but only provided information regarding the orientation of the applied virtual loads at the base of the button. The discretized geometry of the coracoid process including the button consisted of tetrahedral finite elements. Continuum MicroMechanics approach was used to calculate the elasticity of the bone structure [17].

The coracoid process was completely fixed at the surface that attaches to the upper part of the neck of the scapula. This corresponds to the commonly used homogeneous Dirichlet boundary condition for the FE analysis [18]. The deformation of the coracoid process due an applied loading of 8 MPa surface load on the button in the direction that is collinear to the direction of suture was computed using the open-source software Kratos (http://www.cimne.com/kratos). Pre- and post-processing were performed using GID. Von Mises stresses were calculated, which is an equivalent stress measure that characterizes failure due to distortion in the material, as previously used in experimental shoulder studies [19].

### Radiological analysis of button placement on the clavicle

In a next step, a randomly chosen series of 40 patients with high-grade AC joint separations that were treated surgically at our unit between 1/2017 and 4/2021 was identified retrospectively. An experienced observer on fellowship level analyzed the standardized X-ray pictures in order to see, if critical placements of drill holes as described by the finite element analysis could be seen in our patient cohort.

**Fig. 3:**
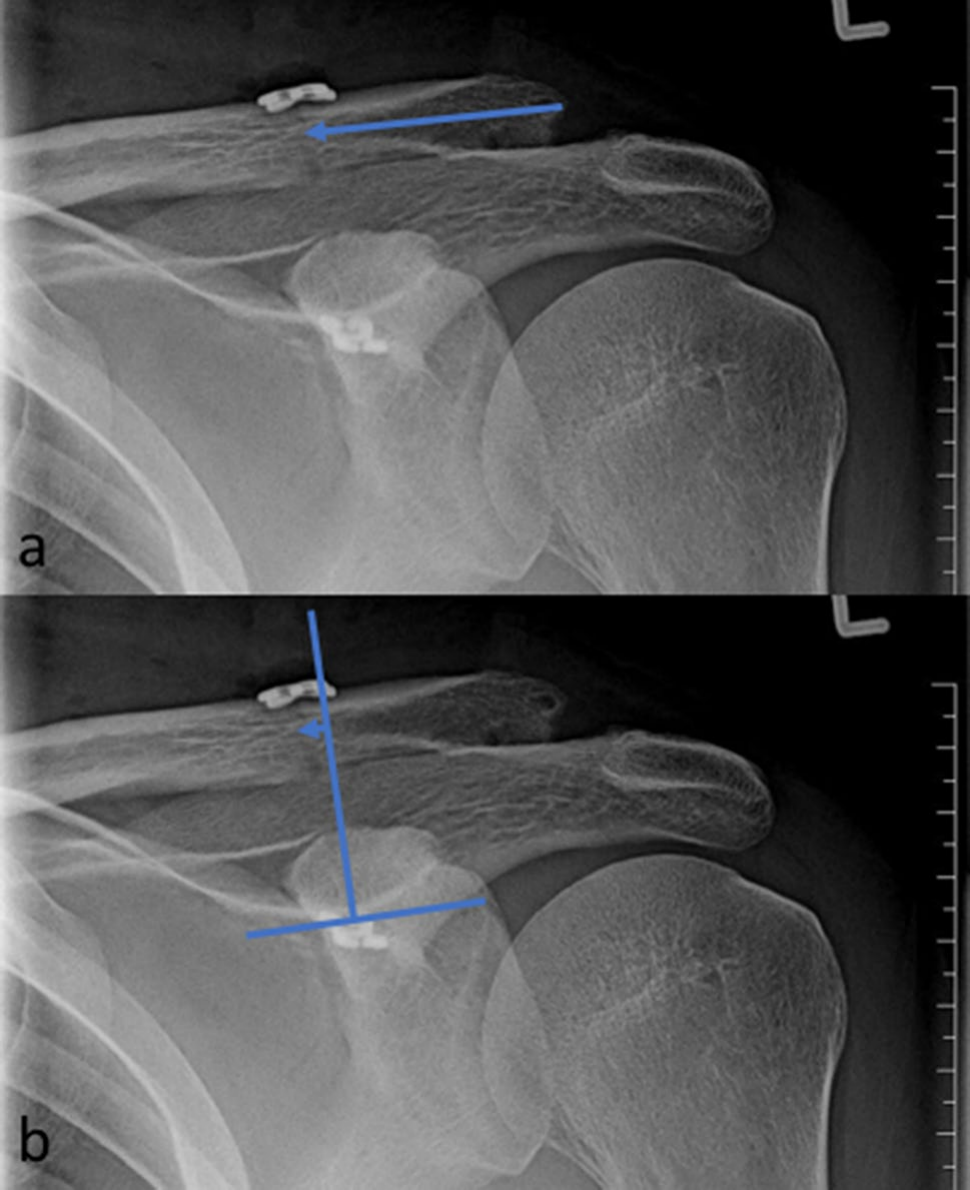
Exemplary radiological analysis in a 38-year-old patient, left shoulder, Rockwood V lesion, 1 day post-surgery: **a** A line was drawn from the lateral edge of the clavicle to the drill hole (CL distance). **b** Then, the distance from a line that was drawn perpendicularly to the coracoid center line and the clavicle drill hole (drill hole placement, DHP). The coracoid center line was referenced by drawing a line beneath the coracoid that was parallel to the undersurface of the clavicle (**b**). If the drill hole was placed medially of the coracoid center, negative values were recorded, and positive values were used for lateral placements

Apart from age and gender, standardized a.p.-radiographs were examined and the following parameters were recorded: grade of injury (according to the Rockwood classification), distance from the lateral edge of the clavicle to the clavicle drill hole (CL distance, Fig. 3a) and the distance from a line that was drawn perpendicularly to the coracoid center line and the clavicle drill hole (drill hole placement, DHP, Fig. 3b). All pictures were obtained on the day after surgery, following a standardized post-operative routine. The coracoid center line was referenced by drawing a line beneath the coracoid that was parallel to the undersurface of the clavicle (Fig. 3b). If the drill hole was placed medially of the coracoid center, negative values were recorded, and positive values were used for lateral placements.

In a next step, the patients were subcategorized into those with an anatomical placement of the button (group ap) and those with an extra-anatomical placement (group eap), considering a CL distance between 29 and 41 mm anatomical, as described by Rios et al. [20].

The measuring results were recorded in a SPSS database (Statistical Package for the Social Science, IBM Cooperation, Armonk, NY, USA). If parameters were normally distributed, analysis of variance (ANOVA) was used in order to compare means. For all other parameters, Mann-Whitney *U* tests were used.

## Results

### Finite element study

**Fig. 4:**
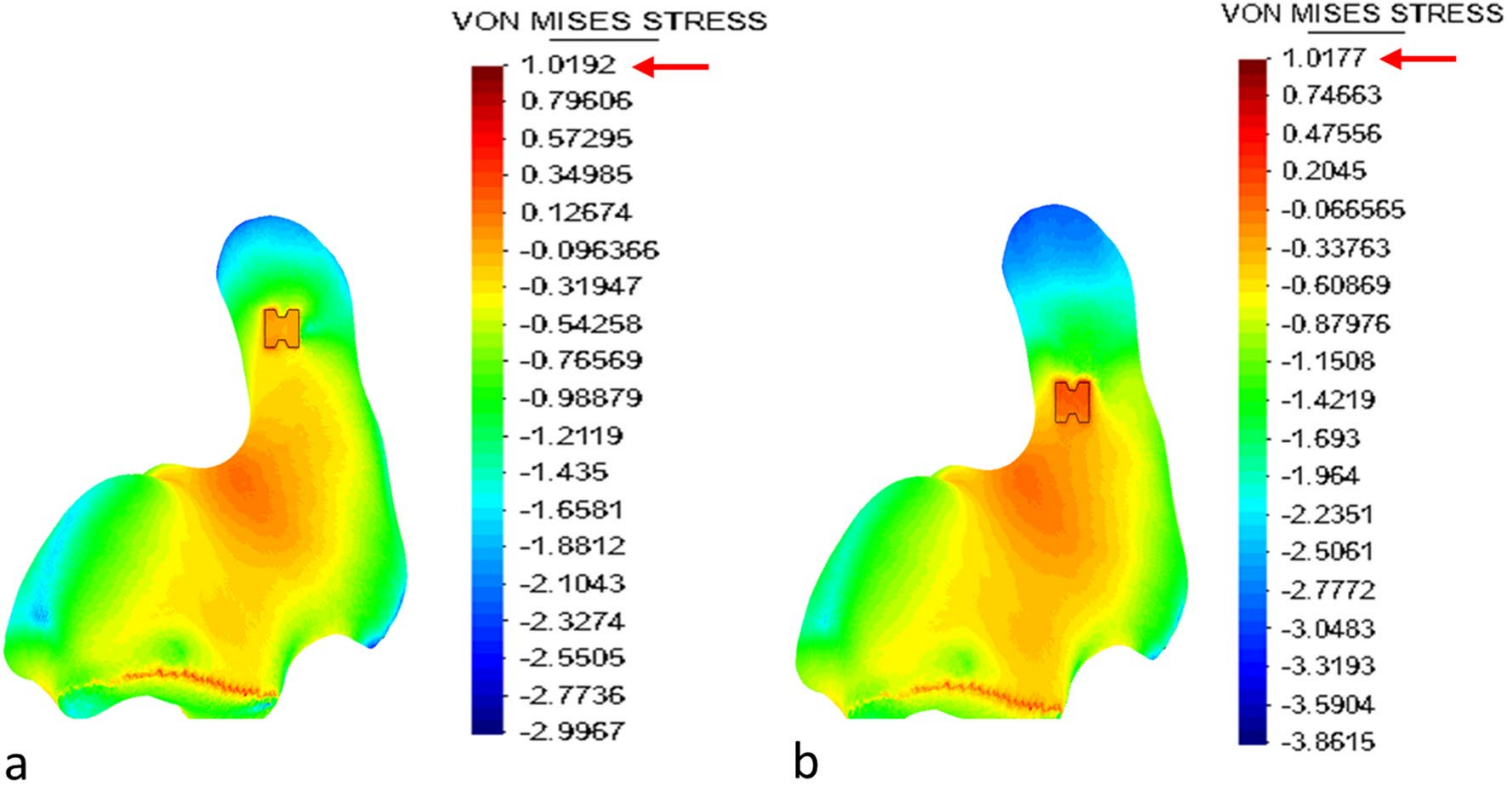
Illustration of the stresses caused by the two most dangerous configurations as analyzed by the von Mises stress analysis (axial view): a distal position of the button with a medial placement of the clavicle drill hole (**a**) and a proximal position of the button with straight traction (**b**). Also see Table 1 for the corresponding numerical values

The results for the maximal stresses recorded can be seen in Table 1 and Fig. 4.

**Table 1:** Maximum stress results for the different tunnel localizations

	Load direction towards clavicle	Max. stress (log scale) (× 10^7^ Pa)	Max. stress (× 10^7^ Pa)
Proximal tunnel in coracoid	Medial	1.0177	10.416
	Central	0.9583	9.086
	Lateral	0.9084	8
Distal tunnel in coracoid	Medial	0.9579	9.077
	Central	1.0192	10.451
	Lateral	0.9813	9.578

× (Log Scale) = 10^*x*^

According to the results of the von Mises analysis, the optimal configuration was a proximal tunnel in the coracoid combined with a lateral bone tunnel in the clavicle (see also Fig. 2 for comparison).

The most dangerous settings, on the other hand, were a distal bone tunnel in the coracoid and perpendicular traction towards the clavicle as well as a proximal tunnel in the coracoid with medial traction towards the clavicle.

Of note, the difference between the maximum and minimum stress configurations was 2.451 × 10^7^ Pa. Thus, using the configuration recommended by our analysis led to stress reduction of 23% compared to the most dangerous setting.

### Radiological analysis

We included 40 patients with a mean age of 42 ± 15 years at the time of injury. 2 patients (5%) were females, 38 (95%) males. In 21 cases (53%), the left shoulder was injured. 5 injuries (13%) were classified as Rockwood 3 lesions, 35 (87%) as Rockwood 5 lesions. Mean CL distance was 29 mm ± 6 mm (group ap: 33 mm ± 3 mm; group eap: 24 mm ± 4 mm; *p* < 0.001), DHP was 6 mm ± 9 mm (group ap: 2 mm ± 7 mm; group eap: 11 mm ± 9 mm; *p* < 0.001). 8 patients (20%) showed a medial placement of the clavicular button; of these, *n* = 7 (88%) were from group ap, and *n* = 1 (12%) from group eap. Mean CL distance of these eight patients was 33 mm ± 5 mm, while DHP was − 4.5 mm ± 2 mm.

## Discussion

The first main finding of our study is that the choice of tunnel positions in the coracoid and lateral clavicle has significant impact on the amount of mechanical stress caused in the coracoid process after CC ligament repair. A plausible reason for that is that the tunnel positions determine the direction in which the traction of the CC repair is aimed.

The second main finding is that anatomical placement of the clavicular button does not prevent dangerous configurations.

Our analysis facilitated a commonly used stress model, the von Mises analysis, which is frequently applied for ductile materials and has a strong emphasis on shear stresses.

There are two possible explanations for our stress results. Concerning the bone tunnel in the coracoid, a proximal localization seems plausible as it leads to a smaller lever arm and, thus, smaller mechanical stress on the basis of the coracoid. However, the bone tunnel should not be drilled at the very base of the coracoid. This would ensure biomechanical stability in theory, but not lead to an anatomical configuration, as the mean distance between the conoid ligament and the base of the coracoid is about 6 mm [21].

This finding is in line with a similar study by Campbell et al., who analyzed different bone tunnel configurations for CC ligament repair in the coracoid [11] and concluded that distal tunnels come with a higher risk of stress fractures, especially when they are not located centrally in the bone.

Concerning the position of the clavicle drill hole (and, thus, the orientation of the traction), the explanation is more complex. The shape of the coracoid has a high variability, as described in previous anatomical studies [22]. In our configuration, a lateral position of the bone tunnel of the clavicle produced the smallest amount of shear stress. Of note, this configuration corresponds to the position of the trapezoid ligament, whose main purpose is to stabilize the AC joint vertically, while the conoid ligament is responsible of the rotational stability of the lateral clavicle. Thus, this tunnel placement could be considered as close to the native anatomy of the vertical AC joint stabilization.

In line with that, an anatomical analysis by Coale et al. argued that an anatomical reconstruction of the CC ligaments can seldomly be considered as a straight line, as the cases analyzed in their 3D CT anatomical study showed an oblique orientation of the CC tunnels [23].

However, anatomical placement of the clavicle drill hole does not prevent medial traction of the overall construct, as shown by our radiological analysis. In the cohort that was analyzed, 20% of the shoulders showed medial traction of the construct and can therefore be considered critical placements. This result can be explained by the great anatomical variability of ac joints, which is caused by many basic factors as gender, body height etc. Thus, the surgeon should keep in mind that the anatomical corridor of 29–41 mm distance from the lateral edge of the clavicle needs to be adapted to the individual patient very carefully.

The authors are aware of the study’s limitations. First of all, a male scapula of a Caucasian patient was utilized. Results may vary for different ethnicities. Secondly, like all experimental studies, our calculations can only be seen as approximations. Thirdly, only one implant was tested. Different results may be expected using other surgical techniques or implants by other manufacturers. Upon that, the clavicle and scapula from two different scans were used. This might affect the results.

## Conclusion

The bone tunnel placement with the smallest amount of shear stresses was found when the traction of the suture button was directed slightly lateral, towards the AC joint. Anatomical placement of the clavicle drill hole alone was not sufficient in preventing dangerous configurations.
